# Design and validation of the presenteeism scale in nursing

**DOI:** 10.1186/s12912-023-01454-y

**Published:** 2023-08-28

**Authors:** Mohammad Mehdi Mohammadi, Nahid Dehghan Nayeri, Shokoh Varaei, Arezoo Rasti

**Affiliations:** 1 Department of Medical Surgical Nursing, School of Nursing & Midwifery, Kermanshah University of Medical Sciences, Kermanshah, Iran; 2 Nursing and Midwifery Care Research Center, School of Nursing and Midwifery, Tehran University of Medical Sciences, Tehran, Iran; 3 Department of Medical Surgical Nursing, School of Nursing & Midwifery, Tehran University of Medical Sciences, Tehran, Iran

**Keywords:** Presenteeism, Nursing, Qualitative Study, Psychometric properties

## Abstract

**Background:**

The instruments used to measure presenteeism are all flawed and only incompletely measure the concept of presenteeism in employees of the general population. As a result, the concept of presenteeism is not measured, and in most of these instruments, the population for which the instrument has been developed differs from the nursing population. The present research was conducted to design and validate the instrument for evaluating presenteeism in nursing.

**Methods:**

The present study was part of an exploratory sequential mixed study. In this study, the instrument for measuring the level of presenteeism among nurses was developed and validated based on the results of the qualitative stage. To this end, the instrument’s psychometric properties were investigated using face, content, and construct validity, as well as reliability through internal consistency and stability.

**Results:**

In this study, an instrument containing 17 items and three dimensions (imperfect cognitive presence, imperfect emotional presence, and imperfect movement presence) with favorable validation characteristics was developed. Therefore, the instrument was able to explain 56.375% of the total variance. Furthermore, Cronbach’s alpha and McDonald’s omega coefficients were 0.881 and 0.815, respectively. The intra-cluster correlation coefficient (ICC) was also reported as 0.972 for the entire instrument, with a 95% confidence interval of 0.941 to 0.987.

**Conclusion:**

Based this study, it was possible to measure the level of nurses’ presenteeism through an instrument with favorable psychometric properties. This study helps health managers lay the groundwork for designing a system for measuring presenteeism among Iranian nurses using the developed instrument.

**Supplementary Information:**

The online version contains supplementary material available at 10.1186/s12912-023-01454-y.

## Background

Presenteeism is the staff’s physical presence at the workplace with reduced performance [1]. In other words, this concept refers to those who are merely physically present at the workplace due to inappropriate conditions (such as illness, poor mental condition, and extreme fatigue) [2]. Based on the definition we already provided regarding presenteeism, this concept in nursing implies the non-actualization of the nurse’s capacities in the arena of presence. In other words, the internal capacities of the nurse in the cognitive, emotional, and movement fields potentially existing in them are not provided with an opportunity to be actualized [3].

Presenteeism among nurses will gradually lead to the destruction of the desired medical organization through its destructive consequences. The medical organization suffering from these unhealthy conditions will lead to increased human errors, decreased productivity and job satisfaction, and various other unknown consequences [4–6]. It's crucial to acknowledge the significance of measuring presenteeism, as its presence often leads to a lack of clinical skills and competencies in healthcare professionals. This can lead to a variety of negative outcomes, such as high turnover rates, decreased job satisfaction, compromised patient safety, and an increased likelihood of medical errors [7, 8]. Therefore, monitoring presenteeism is key in identifying areas where additional support or training may be necessary to improve the quality of care provided by healthcare professionals [9]. Measuring presenteeism in nursing is vital as it helps identify its occurrence in a profession focused on valuable human health. This measurement creates awareness of attendance within the healthcare system and aids in preventing potential errors. Neglecting to measure presenteeism accurately can have harmful consequences for both nurses' well-being and patient care. By accurately assessing and addressing presenteeism, healthcare organizations can foster a healthier work environment and ensure high-quality care for patients [10, 11].

Most instruments to measure presenteeism establish incomplete conceptual compatibility with the main concept. In other words, instead of directly measuring the concept of presenteeism, they indirectly measure its few consequences [12–15]. Moreover, in the majority of these instruments, the target population consists of industrial and administrative employees. Obviously, the employees introduced as factory workers or office employees have entirely different characteristics compared to nurses, and utilizing the instruments designed for these groups, is not considered suitable for nurses [13, 16]. Furthermore, these instruments have been designed for other countries’ contexts and organizational settings; nevertheless, presenteeism is a context-dependent concept influenced by the context in question and necessitates creating an instrument appropriate for the Iranian context [17]. Additionally, the design and psychometric stages of the existing instruments are methodologically flawed, and in the reviews on these instruments, the issues, such as lack of content validity assessment, failure to use factor analysis, defects in measuring the reliability, and numerous methodological criticisms are raised [18].

In the search to identify instruments measuring presenteeism, we came across the Work Limitations Questionnaire (WLQ). Evidence indicates that this instrument has mainly been used to measure employees’ presenteeism [19]. This instrument was introduced in 2001 to measure the impact of employees’ chronic diseases on their productivity. The target population of this instrument consisted of workers suffering from chronic diseases, such as asthma. This questionnaire predominantly focuses on work limitations for industrial workers and is not an appropriate criterion for measuring presenteeism among nurses [12]. Another issue is the nature of the concept of presenteeism among nurses, which is influenced by the organizational and structural context and necessitates the development of a specific instrument for that context.

Another instrument was the Work Productivity and Activity Impairment (WPAI) scale, developed in 1993 to investigate employees’ productivity and performance [15]. Similar to the previous instrument, this instrument was not designed to measure the concept of presenteeism and did not integratively and accurately measure this concept in nursing.

Endicott Work Productivity Scale (EWPS) was another instrument used for this purpose. This instrument was developed in 1997 in a population of depressed patients, and its primary application was to measure productivity in patients with depression. This instrument is used in clinical trial studies in the field of psychiatry in order to investigate the effect of psychiatric interventions on depressed patients’ work productivity [13, 19].

One other instrument was the Stanford scale, developed in 2002. Melancon et al. state that this instrument is invalid for measuring presenteeism and lacks the ability to measure this concept. On the other hand, this instrument has likewise been designed with an emphasis on measuring performance and productivity in the general population with at least a high school education and does not investigate and measure the concept of presenteeism in nursing [14, 20].

Baris et al. conducted a study entitled "Development and psychometric validation of the Sickness Presenteeism Scale-Nurse". However, it is important to note that the tool developed in this study may not be suitable for the context and culture of Iran. A critical review of the study indicates that cultural factors and contextual differences can influence the manifestation and perception of presenteeism among Iranian nurses. Therefore, there is a need to develop a new tool that is specifically tailored to the Iranian context. Such a tool would accurately measure presenteeism among nurses in Iran, capturing the unique aspects and nuances of presenteeism in the Iranian healthcare setting. This would ensure that the data collected from such a measurement tool are more accurate and reliable, which would be beneficial for research and policy-making purposes [21].

In addition, these instruments have been criticized regarding methodological quality; in most cases, the criticisms are so deep that critics, including Ospina, Thompson, and Nuben, in their independent studies, believe presenteeism instruments suffer a crisis in the quality of methodology. Accordingly, issues such as failure to measure content validity, construct validity, internal consistency, and test–retest reliability are evident in these instruments [18, 22].

According to what has been stated, the instruments used to measure presenteeism are defective, and they merely incompletely measure the concept of presenteeism among employees of the general population. As a result, this concept is not measured, and in most instruments, the population for whom the instrument has been designed is not trained in nursing. On the other hand, the concept of presenteeism is affected by the context, and the administrative, cultural, and social background of Iran’s medical organizations vary from many western countries where these instruments have been developed. Consequently, the present research was conducted to design and validate the presenteeism instrument in nursing.

## Methods

### Aim

To design and validate presenteeism scale in nursing.

Research questions:

1. Does the presenteeism scale in nursing have validity in terms of face, content, and construct?
2. Does the presenteeism scale in nursing have reliability in terms of internal consistency and stability?

### Study design and item generation

The present study was a quantitative part (instrument development and validation) of an exploratory sequential mixed study through which an instrument for measuring the rate of presenteeism among nurses was developed and validated from August 2021 to February 2023 based on the results of the qualitative phase. In this study, the inductive-deductive method was used to generate items [23]. To this end, items were extracted from the qualitative phase (inductive). Afterward, the texts were revised for additional items (deductive), and a pool of items was formed. Subsequently, being reviewed by the research team, overlapping and additional items were merged or removed.

The instrument was validated based on the Classical Test Theory (CTT) [24]. To this end, the instrument’s face, content, and construct validity, as well as reliability, were assessed.

### The study sample

In the present study, the main research samples consisted of nurses employed in various departments, including emergency, special care, oncology, internal medicine, surgery, and psychiatric, at the teaching hospitals of Tehran University of Medical Sciences. These nurses had a minimum of one year of work experience and were directly involved in patient care.

### Face validity

#### Determining qualitative face validity

At this stage, ten nurses’ corrective feedback regarding each of the items of the instrument in terms of difficulty (difficulty in understanding phrases and words), irrelevancy (the possible inconsistency of the items with the instrument dimensions), and ambiguity (the possible misperceptions of phrases or insufficient meaning of words) were collected, revised, and modified. In this regard, the population for whom the instrument was developed (nurses) was selected as the participants in this stage.

#### Determining quantitative face validity

After establishing the face validity qualitatively and modifying the items based on the subjects’ opinions, in the next step, the Item Impact Method was used to determine the face validity quantitatively. For each item, a 5-point Likert scale (completely appropriate = 5, appropriate = 4, moderately appropriate = 3, inappropriate = 2, completely inappropriate = 1) was considered. Ten nurses rated each of the instrument items based on the described Likert scale, and the “Item Impact Score” was calculated for each item separately based on the following formula:

$$Item\,Impact\,Score=Frequency\,\left(\%\right)\times Suitability\,\left(Importance\right)$$

In this formula, Frequency was the percentage of the participants who scored the item as 4 or 5, and Suitability was the mean of the scores individuals considered for the item. A score of 1.5 and above indicated the item’s appropriateness, and items with a score of less than 1.5 needed to be revised and modified.

### Content validity

Qualitative and quantitative methods were used to assess content validity [25, 26]:

#### Determining qualitative content validity

In order to establish the qualitative content validity in the present study, ten faculty members experienced in the field of nursing management studies and instrumentation were requested to express their corrective views after revising the instrument meticulously by emphasizing grammar, wording, item allocation, scaling method, simplicity, and clarity.

#### Determining quantitative content validity

In order to quantitatively evaluate the content validity of the instrument, the Content Validity Ratio (CVR) and Content Validity Index (CVI) were used based on the Modified Kappa Statistic (K*). To this end, CVR was measured, and after evaluating the results and removing some items, CVI was examined based on the K* statistic.

#### Content Validity Ratio (CVR)

After applying modifications related to qualitative content validity, the intended instrument entered the CVR stage. The present study involved several steps in calculating the CVR. Firstly, the purposes of the instrument and operational definitions were explained. Secondly, ten experts (faculty members with experience and history in the field of nursing management and instrumentation) were requested to determine the necessity of each item on a 3-point Likert scale. The options included "necessary," "useful but not necessary," and "not necessary." Thirdly, the CVR was calculated using the following formula [27, 28]:

$$CVR=\frac{{n}_{e}-\left(N/2\right)}{N/2}$$

In this formula, *ne* was the number of experts who chose the ‘necessary’ option, and *N* was the total number of experts. After calculating the CVR, with 95% confidence, the minimum acceptable numeric value for CVR was considered 0.62 using the Lawshe Table and based on the number of experts (ten individuals). Accordingly, items with CVR less than 0.62 were excluded, and the remaining items were examined for CVI based on the K* statistic [29].

#### Content Validity Index (CVI)

In order to calculate CVI in the present study, the modified Kappa statistic, which is symbolized by K*, was used [25, 29]. The process of calculating K* involves calculating the I-CVI for each item using a formula proposed by Waltz and Basel. A panel of 12 experts with experience in instrumentation, management, and nursing evaluated each item based on its relevancy using a 4-point Likert scale. The I-CVI was calculated by determining the proportion of experts rating an item as 3 or 4 out of the total number of experts.

$$\mathrm{CVI}= \frac{\text{number of raters giving a rating of }\mathbf{^{\prime}}3\mathbf{^{\prime}}\mathrm{ or }\mathbf{^{\prime}}4\mathbf{^{\prime}}}{\text{total number of raters}}$$

In the second step, the Probability of Chance (Pc) was calculated using the following formula:

$${P}_{C}=\left[\frac{N!}{A!\left(N-A\right)!}\right]\times {0.5}^{N}$$

It should be noted that in this formula, A is the number of experts rating the item as 3 and 4, and N is the total number of experts.

Finally, the adjusted Kappa statistic (K*) was calculated using the following formula:

$${K}^{*}=\frac{I-CVI -Pc}{1-Pc}$$

According to Polit, the basis of judgment based on K* is as follows: obtaining a consensus score of 0.4 to 0.59 = poor, 0.6 to 0.74 = good, and above 0.74 = excellent [27, 28]. Accordingly, in the present study, a result greater than 0.74 was considered a relevant and excellent item.

In addition, in this study, the Scale-Content Validity Index (S-CVI) was calculated based on the mean I-CVIs (S-CVI/Ave). The minimum numeric value of 0.9 was considered the criterion of S-CVI/Ave acceptance.

### Determining initial reliability

Prior to determining construct validity, the initial reliability of the instrument was measured. For this purpose, to check the instrument’s internal consistency (based on Cronbach’s alpha method), 30 nurses working in teaching hospitals in 'REDACTED' were selected through the convenient method. In order to perform an item analysis, the Loop method and Corrected Item Total Correlation were used. To this end, the amount of alpha change was investigated in case the item was removed. It should be noted that if the Corrected Item Total Correlation was less than 0.3, the item was removed [25].

### Construct validity

In the present research, exploratory factor analysis was used to determine the construct validity. For this purpose, the designed instrument was provided online to the target group (nurses). In order to collect the data in this stage, an electronic questionnaire was used. The first section consisted of demographic information, and the second included instrument items on a 5-point Likert scale (always/often/sometimes/rarely/never). This instrument enjoyed a web-based design, and the related link was sent to the participants through social networks. In determining the appropriate sample size for factor analysis, various guidelines have been proposed in the literature. One commonly cited guideline is the Rule of 300, which suggests that there should be at least 300 cases [30]. Another approach proposed by Comrey and Lee categorizes sample sizes as poor (100), fair (200), good (300), very good (500), and excellent (1,000 or more) based on their suitability for factor analysis [31]. In line with these recommendations, we followed the Rule of 300 and selected a sample of 300 participants for our factor analysis., and due to the possible sample attrition based on the exclusion criteria, 320 samples were enrolled in the study. The convenience sampling method was used, and inclusion criteria included being employed in a hospital and directly involved in patient care. The exclusion criteria were the participants who were considered indifferent respondents, and the standard deviation of their responses was less than 0.2. Besides, the univariate and multivariate outliers were cautiously considered candidates for exclusion from the study.

In order to perform exploratory factor analysis, SPSS software version 16 was used. In the first stage, in order to examine the sampling adequacy, Bartlett’s Tests of Sphericity and Keyser-Meyer-Olkin’s test were used. In addition, Kaiser–Meyer–Olkin’s sampling adequacy test was investigated. An index greater than 0.5 was acceptable, although a value higher than 0.7 was considered ideal. Therefore, in the present study, the minimum acceptable level for this index was considered 0.7 [25].

In the present study, the factor extraction method based on the main axis was used to extract the factors. In order to determine the number of factors extracted, the preliminary investigation was performed based on the eigenvalue and scree plot, and the final decision was made based on the Parallel Analysis (PA). Based on the eigenvalue, only factors with an eigenvalue greater than one were examined, and other factors with an eigenvalue less than one were ignored. In the present study, the 95% confidence interval for each eigenvalue was calculated based on the following formula [25, 27, 28]:

$${l}_{i}\pm {z}^{*}\left(\sqrt{\frac{{2l}_{i}^{2}}{n}}\right)$$

In this formula, *li* is the eigenvalue value, and *n* is the sample size. Moreover, if the 95% confidence interval is calculated, *z* will be equal to 1.96. By calculating the confidence interval for each eigenvalue with 95% confidence, it is possible to determine the eigenvalue to be equal to or greater than 1.

In this study, the screen plot was drawn based on the 95th percentile of the simulated data using JASP software, version 0.14. Based on this diagram, the extracted factors are those with an observed eigenvalue greater than the 95th percentile of the simulated data. In this study, in order to perform parallel analysis, Syntax provided by Brian Connor was used in SPSS software version 16 [32].

In this study, after examining different rotations, the Varimax rotation, one of the orthogonal rotations, was selected to clarify the factor construct. Finally, the factors were labeled based on the items of each factor after extraction [33]. Besides, in order to determine the minimum acceptable factor load to maintain each item in the factor, the formula CV = 5⋅152 ÷ √ (*n* − 2) was used at the 99% confidence level. In this formula, *n* is the sample size [34]. Based on this, the minimum acceptable factor loading in the present study was calculated to be approximately 0.3.

### Identifying indifferent respondents

In the present study, in order to identify and eliminate indifferent respondents, the standard deviation of each participant’s responses was used. To this end, respondents whose answers had a standard deviation of less than 0.2 were excluded from the study. It should be mentioned that this process was carried out through Excel software version 2007 and function writing according to standard deviation.

### Investigating the normal distribution of the data and univariate and multivariate outliers

The normality of data distribution was evaluated based on the skewness (± 3) and kurtosis (± 7) indices, and univariate outliers were identified using a Box plot. The cases identified as outliers in most variables were a possible option to be removed from the data. To identify multivariate outliers, the Mahalanobis d-square test was performed using Amos software version 25. Items with P < 0.001 were considered as a possible option for exclusion.

### Ultimate reliability

The present study assessed internal consistency and stability using Cronbach's alpha coefficient and test–retest method, respectively. Cronbach's alpha above 0.7 was deemed acceptable, with Average Inter-Item Correlation (AIC) and McDonald's omega (ω) being reported as well. McDonald’s omega was calculated using the Omega = 1- [(N-SUM h*2) ÷ (N + 2r)] formula, in which N is the number of items, Sum h*2 is the sum of items shared, and r is the total item loadings. AIC's optimal level was considered between 0.15 and 0.5, while the acceptable omega was greater than 0.7 [35, 36]. Cronbach’s alpha coefficient and AIC were calculated by 30 participating nurses, and Macdonald’s Omega index was calculated using exploratory factor analysis results.

For the test–retest method, 30 nurses completed the developed instrument twice, with a two-week gap. The Intra-Cluster Correlation Coefficient (ICC) was calculated using a two-way mixed model and based on Absolute Agreement, with an ICC of 0.75 and higher being suitable [37]. The appropriate sample size was calculated to be 30, using Power Analysis with an alpha of 0.05 and power of 0.8 through PASS software version 11 [38].

The intra-cluster correlation coefficient alone provides the relative reliability of the instrument [25]. In order to determine the absolute reliability, in addition to reporting this coefficient, the Standard Error of Measurement (SEM) was also calculated based on the following formula:

$$SEM={SD}_{Pooled}\times \sqrt{(1-{ICC}_{agreement})}$$

It should be noted that in this formula, *SD pooled* is calculated using the formula *SD Pooled* = (SD1 + SD2) /2.

### Determining floor and ceiling effects

In this study, the floor and ceiling effects were calculated based on the percentage of respondents obtaining the lowest or highest possible score, respectively. Moreover, the criterion for the presence of floor and ceiling effects in this study was considered to be at least 15%.

### Weighting of items

In the present study, the weighting of items was performed based on the factor analysis results. All steps are provided in Appendix 1. Since the weighting of the items reduces the ease of instrument use, this study dealt with a statistical comparison, “weighting based on factor analysis,” and the “fixed weights equal to one” approaches. To this end, if no statistically significant difference is observed with the help of Friedman’s test, the “fixed weights equal to one” approach, which is naturally more straightforward, is introduced; otherwise, the weighting of the items based on factor analysis will be the preferred method.

### Scoring the instrument

In this study, in order to score the instrument, a method known as Simple Linear Transformation was used [25]. Using this method, the possibility was provided to standardize the score obtained by the respondents out of 100. The reason for this conversion was to facilitate mental approximation and interpretability of the score based on 100. In order to achieve this goal in the present instrument, first, the range of the total scores was calculated based on the 5-point Likert scale. Afterward, based on the following formula, linear transformation of the obtained scores was performed:

$$\frac{raw\,score\,-\,minimum\,instrument\,score}{maximum\,instrument\,score-minimum\,instrument\,score}\times\,100$$

The obtained score ranged between 0 and 100, and a higher score indicated more presenteeism.

### Findings

The initial version of the instrument consisting of 30 items was developed using the inductive-deductive method. After merging overlapping items and removing unrelated ones, the research team created a pool of 48 items that were later reduced to 30 items.

Face validity was determined through qualitative and quantitative methods. Seven items were modified based on interviews with ten nurses to improve their clarity. All items had an item impact score above 1.5, indicating their appropriateness from the respondents' perspective.

Content validity was also determined qualitatively and quantitatively. After interviewing ten faculty members, three items were modified and revised. The Content Validity Ratio (CVR) stage removed seven items due to scoring less than 0.62, leaving 23 items to be assessed for CVI. Based on the K* statistic, two more items were removed, and the instrument entered the reliability determination stage with 21 items. The entire instrument's content validity index was reported as S-CVI/Ave = 0.98.

The initial reliability of the instrument was established using Cronbach's alpha coefficient, which was reported as 0.889. Removing any item did not significantly improve the coefficient. Additionally, the Corrected Item Total Correlation was investigated, and no item reported a correlation lower than 0.3, making no item a candidate for elimination. With 21 items remaining, the instrument entered the construct validity stage.

The study used exploratory factor analysis to determine the construct validity of the presenteeism instrument for nursing, with an initial sample size of 320 nurses. After removing indifferent participants and outliers, the final sample size was 302 individuals, with a mean age of 37.36 ± 5.54 years. Of the participants, 46% were male and 54% were female, while 52% were married and 48% were single. The mean work experience was 12.03 ± 5.54 years.

**Fig. 1:**
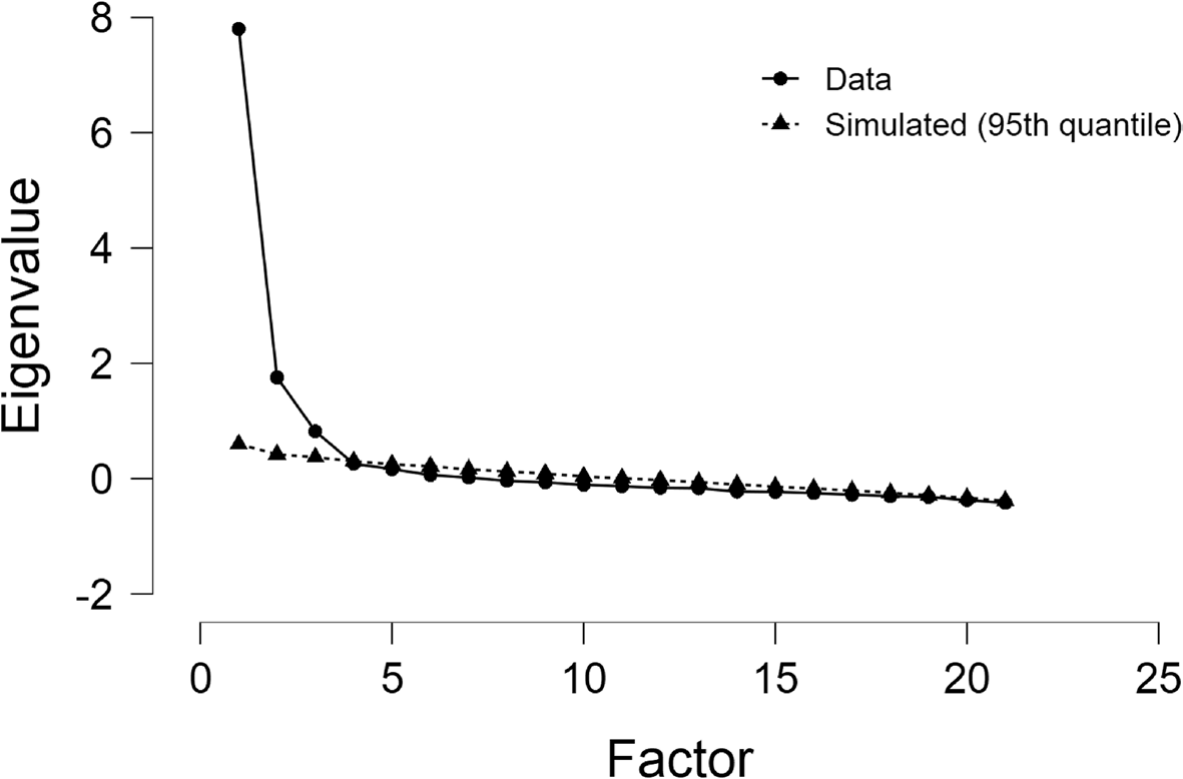
Scree plot to determine the number of tool components

The KMO index was estimated at 0.928, indicating excellent sampling adequacy, and Bartlett’s sphericity test was significant (*P* < 0.0001). The Screen Plot showed visually that three factors could explain the factor construct of the instrument (Fig. 1). Parallel analysis confirmed that these three factors were significant and maintained, with eigenvalues greater than the 95th percentile of simulated data (Table 1). The final instrument consisted of 17 items, explaining 56.375% of the total variance (Table 2).

**Table 1:** Parallel analysis results based on observed and random eigenvalues

Factor	Observed eigenvalue (95% confidence interval)	Random eigenvalue
1	8.405 (7.9–064.746)	1.451
2	2.388 (1.1–144.581)	1.361
3	1.431 (1.1–09.51)	1.297
4	0.899 (0.1–756.043)	1.234
5	0.830 (0.0–698.963)	1.181
6	0.754 (0.0–634.874)	1.132

**Table 2:** Eigenvalues and explained variance for the extracted factors

Factor	Initial Eigenvalues			The sum of the squares of the extracted factor loadings			The sum of the squares of the extracted factor loadings		
	Total	Variance	The cumulative percentage	Total	Variance	The cumulative percentage	Total	Variance	The cumulative percentage
1	7.327	43.098	43.098	6.917	40.686	40.686	3.639	21.406	21.406
2	2.143	12.609	55.707	1.699	9.993	50.680	3.230	19.003	40.408
3	1.319	7.760	63.466	0.968	5.696	56.375	2.714	15.967	56.375
4	0.870	5.121	68.587						
5	0.700	4.116	72.703						
6	0.654	3.850	76.553						

The minimum acceptable factor loading was almost 0.3 based on the sample size of 302, and all factor loadings were above 0.5 after varimax rotation. The highest factor loading was 0.840, and the lowest was 0.542 (Table 3).

**Table 3:** Extracted factor loadings after Varimax rotation

Item number	Items	Factor loading		
		Factor 1	Factor 2	Factor 3
1	I am distracted and not focused at work	0.587		
2	I am struggling to remember the patient’s clinical information	0.629		
4	I have delays in making clinical decisions	0.574		
5	I am unable to prioritize my clinical tasks regarding their importance	0.652		
6	At the workplace, my mind is engaged with issues other than patient care	0.703		
7	I lack concentration in my work, so I may repeat specific care	0.671		
8	I do my work duties slower than usual due to mental engagement	0.667		
9	I forget important principles in clinical care	0.542		
12	When I’m at work, I feel like I’m a programmed, soulless robot		0.621	
13	I am unable to provide effective, compassionate care		0.741	
14	I am unable to understand the patient’s vulnerability, suffering, and sadness		0.793	
15	At the workplace, a poker face, a faint smile, and no emotion are manifested in me		0.680	
16	I do not feel dynamic and cheerful at the workplace		0.746	
18	I lack the previous physical ability to perform clinical skills			0.745
19	I lack the physical ability to stand for a long time to do my duties			0.840
20	I feel pain in certain physical positions (bending the neck down to write a file, etc.)			0.714
21	I am challenged in independently performing clinical skills that are individual in nature			0.682

Factors were labeled based on the constituent items, as described in Table 4. To determine the instrument's reliability, several statistics were calculated. The Cronbach's alpha coefficient for the entire instrument was found to be 0.881, indicating high internal consistency. Furthermore, the Average Inter-item Correlations (AIC) and McDonald's Omega were calculated for the entire instrument, with values of 0.337 and 0.815, respectively. The coefficients for each factor are separately reported in Table 5.

**Table 4:** Factor labeling and description

Factor’s name	Factor description
The first factor: **imperfect cognitive presence**	The first factor, which includes eight items, explains 21.406% of the total variance. These items refer to issues such as lack of concentration, distraction, and inability to prioritize tasks. Therefore, this factor was labeled as “imperfect cognitive presence.”
The second factor: **imperfect emotional presence**	This factor explains 19.003% of the total variance of the instrument. The five items of this factor refer to issues such as loss of compassionate care, lack of understanding of the client’s vulnerability, and loss of emotions and sense of dynamism and vitality at the workplace. Accordingly, this factor was labeled as “imperfect emotional presence.”
The third factor: **imperfect movement presence**	The third factor explains 15.967% of the total variance of the instrument. This factor, including four items, refers to the issues such as loss of former physical strength, pain in certain physical positions, and inability to stand for a long time. Therefore, this factor was labeled as “imperfect movement presence.”

**Table 5:** Cronbach’s alpha, Macdonald’s omega, and Average Inter-item Correlations (AIC) by each factor

Average inter-item correlation (AIC)	McDonald’s omega (ω)	Cronbach’s alpha coefficient (α)	Number of items	Factor
0.475	0.759	0.870	8	The first factor: **imperfect cognitive presence**
0.481	0.853	0.812	5	The second factor: **imperfect emotional presence**
0.474	0.870	0.791	4	The third factor: **imperfect movement presence**
0.337	0.815	0.881	17	The entire instrument

The test–retest method was used to determine stability, with an Intra-cluster Correlation Coefficient (ICC) of 0.972 for the entire instrument, with a 95% confidence interval of 0.941 to 0.987. The standard error of measurement for the entire instrument was estimated to be 1.411 (Table 6).

**Table 6:** Intra-cluster Correlation Coefficient (ICC) by each factor

Factor	Number of items	Intra-cluster correlation coefficient (ICC)	95% confidence interval		Standard error of measurement
			high	low	
The first factor: **imperfect cognitive presence**	8	0.990	0.995	0.979	0.361
The second factor: **imperfect emotional presence**	5	0.963	0.982	0.921	0.924
The third factor: **imperfect movement presence**	4	0.926	0.846	0.965	0.912
The entire instrument	17	0.972	0.987	0.941	1.411

Floor and ceiling effects were calculated for the entire instrument and each factor, but were not evident in all cases (Table 7). The item weights were determined by assigning the highest and lowest percentage of variance to the first and third factors, respectively, and then calculating the ratio of the second value of each item to the total second values (Table 8).

**Table 7:** Floor and ceiling effects by factors

Factors	Number of items	Floor effect (percentage)	Ceiling effect (percentage)
The first factor: **imperfect cognitive presence**	8	1.7	0.3
The second factor: **imperfect emotional presence**	5	4.6	0.7
The third factor: **imperfect movement presence**	4	5.3	1.3
The entire instrument	17	1.7	0.3

**Table 8:** Determining the weight of items based on the results of factor analysis

	Items	Factors			Second values	The weight of each factor
		1	2	3		
		The ratio of the variance of each factor to the total variance				
		37.971	33.708	28.322		
1	I am distracted and not focused at work	0.587			22.289	6
2	I am struggling to remember the patient’s clinical information	0.629			23.901	6
3	I have delays in making clinical decisions	0.574			21.787	6
4	I am unable to prioritize my clinical tasks regarding their importance	0.652			24.771	6
5	At the workplace, my mind is engaged with issues other than patient care	0.703			26.681	7
6	I lack concentration in my work, so I may repeat specific care	0.671			25.482	6
7	I do my work duties slower than usual due to mental engagement	0.667			25.326	6
8	I forget important principles in clinical care	0.542			20.571	5
9	When I’m at work, I feel like I’m a programmed, soulless robot		0.621		20.295	5
10	I am unable to provide effective, compassionate care		0.741		24.986	6
11	I am unable to understand the patient’s vulnerability, suffering, and sadness		0.793		26.717	7
12	At the workplace, a poker face, a faint smile, and no emotion are manifested in me		0.680		22.905	6
13	I do not feel dynamic and cheerful at the workplace		0.746		25.159	6
14	I lack the previous physical ability to perform clinical skills			0.745	21.106	5
15	I lack the physical ability to stand for a long time to do my duties			0.840	23.779	6
16	I feel pain in certain physical positions (bending the neck down to write a file, etc.)			0.714	20.228	5
17	I am challenged in independently performing clinical skills that are individual in nature			0.682	19.315	5

The statistical analysis results based on the Friedman test indicate a significant difference in ranking between the two weighting approaches, 'fixed weights equal to one' and 'weighting items using factor analysis', with observed differences leading to rankings in both approaches (*P* < 0.001). Therefore, the preferred method is weighting items based on factor analysis.

The presenteeism instrument in nursing includes 17 items within three dimensions (see Appendix 2). Responses are scored on a 5-point Likert scale ranging from never [1] to always [5]. Each item's score is obtained through multiplying the weight of that item by the score obtained on the Likert scale. The final score is obtained using a linear transformation formula (Table 9) and ranges from zero to 100, with higher scores indicating greater presenteeism.

**Table 9:** Scoring the instrument of presenteeism in nursing ${}^{1}\!\left/ \!{}_{7}\right.$ ${}^{1}\!\left/ \!{}_{7}\right.$

Dimensions	Item weight	Number of items	The range of scores of each item after applying the weight of the item	The range of scores of dimensions after applying the weight of the item	Calculation of the score according to the 0–100 scale (linear transformation of the score)
Imperfect cognitive presence (8 items)	5	1	5–25	48–240	$\frac{\mathrm{score}-48 }{192}\times 100$
	6	6	6–30		
	7	1	7–35		
Imperfect emotional presence(5 items)	5	1	5–25	30–150	$\frac{\mathrm{score}-30 }{120}\times 100$
	6	3	6–30		
	7	1	7–35		
Imperfect movement presence (4 items)	5	3	5–25	21–105	$\frac{\mathrm{score}-21 }{84}\times 100$
	6	1	6–30		
The entire instrument (17 items)			Range of scores:99–495		$\frac{\mathrm{score}-99 }{396}\times 100$

## Discussion

In the present study, the design and validation of the instrument led to the development of the nursing presenteeism instrument containing three dimensions and 17 items. This instrument was designed and validated to measure the rate of nurses’ presenteeism.

The first dimension of the current tool was *imperfect cognitive presence*, which accounted for 21.406% of the total variance and consisted of eight items. It is important to note that this dimension explained the highest percentage of the total variance compared to other dimensions in the instrument. The items within this dimension included distractions and lack of concentration, delays in decision-making, poor task prioritization, forgetting important clinical care principles, mental concerns, and engagement of the mind.

In the Endicott Work Productivity Scale (EWPS) developed by Endicott et al., two items—"at work, you forget to contact other units of the factory" and "at work, you forget to respond to requests from the production manager"—were relatively consistent with some items in the present tool's *imperfect cognitive presence* dimension. In contrast, our tool included forgetfulness in the clinical field, such as " I forget important principles in clinical care" and "I am struggling to remember the patient’s clinical information". Endicott et al. specifically addressed workplace forgetfulness among industrial workers without considering bedside nursing care in their questionnaire [13].

Another item mentioned in the Endicott's tool aligned with the current tool was "I don't focus on my work duties at work," which could be combined with items from our tool like "I am distracted and not focused at work" and "I lack concentration in my work, so I may repeat specific care"; Therefore, our tool covered the lack of concentration more comprehensively than Endicott's tool [13].

In the Lerner et al. work limitations questionnaire, we found the item "At work, I don't focus my mind on my work," which was aligned with the concentration-based items in our tool. However, none of the tools used in presenteeism research addressed delays among nurses' decision-making, poor prioritization of clinical tasks, repetition of clinical tasks, or slowness in performing clinical tasks, and none of them addressed bedside nursing care [16].

Existing tools only focused on office spaces, workshops, and industrial factories, where workers interact with machines, attend meetings, and engage with production units. However, they did not address the unique human interactions between nurses and patients at the bedside.

The present tool's second dimension was *imperfect emotional presence*, and it consists of five items that explain 19.003% of the instrument's total variance. This dimension indicates that nursing professionals may lack dynamism and vitality in their work. When nurses show up to work with a masked face, lost smile, and withered emotions, they resemble programmed, soulless, emotionless robots who merely complete tasks without considering the human aspect of the nursing profession. As a result, compassionate care cannot be provided effectively, and nurses might not understand patients' vulnerability, suffering, and grief accurately. Kim and colleagues argue that presentism translates to a loss of the spirit of nursing, where nurses do not have the chance to manifest their caring hearts, leading to excessive spiritual-emotional fatigue that eventually leads to demoralization of the care provided [39].

This dimension of the present tool appears unique to the nursing profession because nursing deals with the human aspect of healthcare, the concepts of feeling, art, tenderness, and spirit of care. It is this emotional presence that distinguishes the nursing profession from other industrial jobs. Industrial workers deal with raw materials, industrial machinery, clients, and other industrial units but not with human beings. Florence Nightingale emphasized that nurses must use their hands, heart, and mind to create an improved and healing environment for patient care. In other words, it is the nurse's heart that covers a special aspect of their work duties that cannot be found in any industry-related jobs [40]. Holistic philosophy is central to the nursing profession, which respects the unique humanity of all people regardless of who they are. This philosophy focuses on human healing, which is absent in industrial jobs [41].

The third dimension of the tool was *imperfect movement presence*. This dimension encompasses issues such as feeling pain in specific physical positions, loss of physical strength, inability to stand for extended periods, and difficulty performing independent skills. None of the presenteeism tools available completely or ideally cover issues related to an individual's physical presence at the workplace. However, Reilly et al. created a tool titled "Work Productivity and Activity Impairment (WPAI)" which examines a limited aspect of an individual's physical performance in life. The tool includes an item that emphasizes physical activities outside the workplace, such as working at home, shopping, taking care of children, and participating in sports [15]. However, this tool does not specifically focus on presenteeism; rather, it measures aspects of quality of life during its psychometrics. The physical presence of a nurse is crucial, along with their cognitive and emotional presence, for an ideal bedside presence. According to Florence Nightingale, nurses should employ not just their hearts and minds but also their hands to establish a better and restorative atmosphere [40]. In this context, a nurse's hands represent their physical presence, while their heart signifies emotional presence, and their mind denotes cognitive presence. These three elements are fundamental to achieving an ideal presence in modern nursing.

The limitation of the present study was the risk of contracting the coronavirus disease during data collection. In order to minimize this risk, an effort was made to use an electronic questionnaire to collect data at the construct validity stage. In other stages of data collection, wearing a mask, maintaining a distance of at least two meters from the participant, and taking into account proper ventilation in the interview location, were considered.

It is recommended that the instrument developed in the present study be used in other studies on nursing. Furthermore, it is advised that an instrument be designed in future studies to determine the causes of presenteeism based on the antecedents of the concept.

## Conclusion

In the present study, an instrument including 17 items and three dimensions was developed that enjoys good validity and reliability and provides the possibility of measuring presenteeism in nurses. The present instrument can help healthcare managers obtain information about the level of presenteeism among nurses since the assessment of the current situation can be the first step in developing management plans related to presenteeism. Moreover, this instrument can be used in a wide range of research related to presenteeism; as a result, the implementation of research related to presenteeism in Iran will be facilitated, and a significant step will be taken in developing and promoting this concept in Iranian research.

### Supplementary Information

**Additional file 1:** **Appendix 1.** The steps of item weighting in this study.

**Additional file 2:** **Appendix 2.** The final version of the presenteeism scale in nursing.

## Data Availability

The data analyzed and materials used in this study are available from the corresponding author on reasonable request.
