# Supplementary material for: Design and validation of the presenteeism scale in nursing
Source: BMC Nurs. 2023 Aug 28;22:290. doi: 10.1186/s12912-023-01454-y (PMC10463333; doi:10.1186/s12912-023-01454-y)
Supplement: Supplementary file 1 — Additional file 1: Appendix 1. The steps of item weighting in this study. [file 12912_2023_1454_MOESM1_ESM.docx]

Appendix 1: The steps of item weighting in this study

| Step | Description |
| --- | --- |
| 1 | First, the variance ratio of each factor to the total explained variance was calculated according to the following formula:  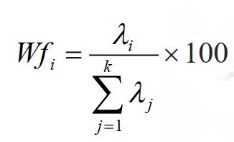  *λ_i:_* The ratio of the variance of the ith factor to the total changes by all items  *Wfi:* The relative percentage of the variance of the ith factor from the total changes of the items  Based on this, each factor’s ratio percentage of the explained variance to the cumulative variance of all extracted factors was calculated separately. |
| 2 | In the next step, the second value for each item was calculated using the following formula:  Second Value= Factor Loading (fi) × W(fi)  To calculate the second value for each item, we multiplied the factor loading of each item by the factor variance ratio of that item (calculated in the first step). |
| 3 | In the next step, we summed up the calculated second values to determine the total second values. |
| 4 | At the end, the weight of each item was equal to the percentage of the second value of each item divided by the total second values:  Item Weight (i) = (Second Value / Total Second Value) × 100  It should be noted that the weight of each item is multiplied by the raw score obtained from the answers in the instrument. |
