# Supplementary material for: Design and validation of the presenteeism scale in nursing
Source: BMC Nurs. 2023 Aug 28;22:290. doi: 10.1186/s12912-023-01454-y (PMC10463333; doi:10.1186/s12912-023-01454-y)
Supplement: Supplementary file 2 — Additional file 2: Appendix 2. The final version of the presenteeism scale in nursing. [file 12912_2023_1454_MOESM2_ESM.doc]

| **Appendix 2**: The final version of the presenteeism scale in nursing | | |
| --- | --- | --- |
| Items | Dimensions |  |
| I am distracted and not focused at work. | Imperfect cognitive presence | 1 |
| I am struggling to remember the patient’s clinical information. |  | 2 |
| I have delays in making clinical decisions. |  | 3 |
| I am unable to prioritize my clinical tasks regarding their importance. |  | 4 |
| At the workplace, my mind is engaged with issues other than patient care. |  | 5 |
| I lack concentration in my work, so I may repeat specific care. |  | 6 |
| I do my work duties slower than usual due to mental engagement. |  | 7 |
| I forget important principles in clinical care. |  | 8 |
| When I’m at work, I feel like I’m a programmed, soulless robot. | Imperfect emotional presence | 9 |
| I am unable to provide effective, compassionate care. |  | 10 |
| I am unable to understand the patient’s vulnerability, suffering, and sadness. |  | 11 |
| At the workplace, a poker face, a faint smile, and no emotion are manifested in me. |  | 12 |
| I do not feel dynamic and cheerful at the workplace. |  | 13 |
| I lack the previous physical ability to perform clinical skills. | Imperfect movement presence | 14 |
| I lack the physical ability to stand for a long time to do my duties. |  | 15 |
| I feel pain in certain physical positions (bending the neck down to write a file, etc.). |  | 16 |
| I am challenged in independently performing clinical skills that are individual in nature. |  | 17 |
